# Change in Rhetoric but not in Action? Framing of the Ethical Issue of Modern Slavery in a UK Sector at High Risk of Labor Exploitation

**DOI:** 10.1007/s10551-021-05013-w

**Published:** 2021-12-07

**Authors:** Gabriela Gutierrez-Huerter O, Stefan Gold, Alexander Trautrims

**Affiliations:** 1 King’s Business School, King’s College London, Bush House, 30 Aldwych, London, WC2B 4BG UK; 2 Institute of Management and Business Studies, University of Kassel, Nora-Platiel-Str. 4, 34109 Kassel, Germany; 3 University of Nottingham’s Rights Lab, Highfield House, Nottingham, NG7 2RD UK

**Keywords:** Modern slavery, Labor exploitation, Ethical issue, Framing, Moral legitimacy, Longitudinal study, Decent work, Construction

## Abstract

This article shows how the ethical framing of the contemporary issue of modern slavery has evolved in UK construction, a sector in which there is a high risk of labor exploitation. It also examines how these framing dynamics have inhibited the emergence of a common framework of action to deal with the issue. We draw on both framing theory and the literature on the discursive construction of moral legitimacy. Our longitudinal analysis reveals that actors seeking to shape the debate bring their own moral schemes to justify and construct the legitimacy of their frames. Actors cluster their views around five evolving frames: human rights issue (later shifting to hidden crime), moral issue, management issue (later shifting to human moral obligation), social justice issue, and decent work issue—which promote particular normative evaluations of what the issue is, who is responsible, and recommendations for action. Our study contributes to a dynamic and political understanding of the meaning making of modern slavery. We identify the antecedents and conditions that have forestalled the emergence of new patterns of action to tackle modern slavery in the UK construction sector thereby evidencing the effects of the interplay of morally competing frames on field-level change.

**Supplementary Information:**

The online version contains supplementary material available at 10.1007/s10551-021-05013-w.

## Introduction

The term “modern slavery” has received heightened attention in contemporary society and become ubiquitous in debates around extreme forms of labor exploitation (Craig et al., 2019). The discourse emerged as part of a series of developments in both international and UK domestic policy, including the Palermo Protocol, the Council of Europe’s 2005 Convention on Action Against Trafficking in Human Beings and the 2015 UK Modern Slavery Act (MSA). On the outskirts of business and management, the concept itself is plagued with controversy (Craig et al., 2019; LeBaron & Pliley, 2021). The term is challenged and contested (Allain, 2012), with no clear agreement on which phenomena, practices, and actors should be subsumed under this label (Quirk, 2011). As an umbrella term for serious forms of labor exploitation, it has been criticized for covering too wide a range of heterogeneous phenomena, including debt bondage, forced labor, and trafficking in persons (O’Connell Davidson, 2015), and for obscuring the dynamics of the global economy that shape workers’ vulnerability to exploitation (Barrientos et al., 2013). Other debates in modern slavery scholarship concern the effectiveness of legal instruments (Balch, 2019; Mantouvalou, 2018; Nolan & Bott, 2018), the success of private labor governance (LeBaron & Lister, 2021), and the misuse and inaccurate use of historical research (Pliley, 2021). Contributions from business and management to these and other debates have been limited (Caruana et al., 2021). Only recently have management scholars started partaking in those conversations (Smith & Jones, 2020), influenced by the seminal work of Crane (2013), who conceptualized modern slavery as a management practice.

The legal obligation of businesses to report on their policies and measures to tackle modern slavery has spurred research on compliance (e.g., Christ et al., 2019; Flynn, 2020; Monciardini et al., 2021; Rogerson et al., 2020; Schaper & Pollach, 2021). Beyond corporate reporting, increasing interest has emerged on the strategic use of modern slavery frames, through the study of discourse and narratives. These studies have focused on understanding how specific groups of business and non-business actors tap ethical and moral argumentative devices (e.g., Christ & Burritt, 2018; Islam & Van Staden, 2021; Meehan & Pinnington, 2021; Vestergaard & Uldam, 2021; Wray-Bliss & Michelson, 2021) to shape the modern slavery debate. What has yet to be examined is how modern slavery frames of interacting actors, have evolved and account for the emergence of a common framework of action, that is, “field settlement” (Furnari, 2018; Litrico & David, 2017) to tackle modern slavery.

To address this gap, we investigate how a variety of actors in the UK construction sector (including businesses, politicians, campaigners, NGOs, unions, professional associations, think tanks, and certification bodies), which we refer to as “field actors,” construct the meaning of modern slavery and hence contribute to the emergence of field settlement. Following similar approaches (Dahan & Gittens, 2010; Wray-Bliss & Michelson, 2021), we conceptualize modern slavery as an ethical issue which meaning is socially constructed in a dynamic manner (Caruana, 2018).

Our theoretical framework draws from an interactional perspective on framing theory (e.g., Gray et al., 2015) and combines insights from the literature on the discursive construction of moral legitimacy (e.g., Palazzo & Scherer, 2006; Scherer et al., 2013). To characterize modern slavery in the UK construction sector, we adopt an issue-field approach (Hoffman, 1999). From this perspective, the issue of modern slavery becomes a political arena where different sets of actors communicatively compete and deliberate over its framing and assign moral legitimacy to their frames; calling upon moral values and principles, they define the problem, assign responsibility to specific parties, and devise potential solutions.

The UK Home Office (2019) has reported that construction is ranked sixth nationally among the industrial sectors with the highest prevalence of labor exploitation. The sector has in fact been heavily criticized for various unethical practices (Shah & Alotaibi, 2018) and provides a prominent and revelatory case (Yin, 2014) of modern slavery framing and an exemplary source of insights for our research question:

How does the ethical framing of modern slavery play a role in the emergence of field settlement over time in a high-risk sector for labor exploitation in the UK?

The research data, drawn from both primary and secondary sources, span the period 2014–2019. We adopt a semi-inductive interpretive analysis and identify the emergence of five frames: human rights issue (later shifting to hidden crime), moral issue, management issue (later shifting to human moral obligation), social justice issue, and decent work issue. Our longitudinal analysis shows how frame trajectories alter over time, with realignments, interactions and changes in frame adherence. By reconstructing these dynamics, we illuminate a path to rhetorical field settlement (Feront & Bertels, 2019) and outline the conditions that gave rise to the emergence of frames that sought to revise the accepted norms and challenge the prevailing moral order in the field. We reveal that the combination of the early dominance of overly rigid frames that failed to assign moral responsibility to businesses, the limited counter-framing directed to dominant business actors, intra-frame disputes among businesses and the construction of a late frame (the “decent work”) that failed to gain sufficient business support, forestalled the emergence of common patterns of action to tackle modern slavery. Our study contributes to a dynamic understanding of the meaning making of modern slavery by showing that frames are not static and are multivocal. Thus, we redirect attention to a plural, fine-grained understanding of the concept as it is constructed and debated by practitioners. The paper also brings focus to the politics of modern slavery frames and framing and exposes that some marginalized voices had been left out of the framing debate while other dominant voices have been disproportionately amplified reinforcing power asymmetries. Finally, we advance business ethics (BE) literature on the discursive construction of moral legitimacy by evidencing the outcomes of the interplay of morally competing frames on field-level change.

## Theoretical Background

### Modern Slavery Research

Modern slavery as a research topic has gained increased attention from various academic disciplines but there have been few contributions from business and management (Caruana et al., 2021). Over the years, modern slavery scholarship has been permeated with controversies (Craig et al., 2019; LeBaron & Pliley, 2021), reflecting the diversity in the epistemological and theoretical roots of each discipline.

One of the key debates—raised by law scholarship—pertains to the definition of contemporary forms of slavery (Allain, 2012; Quirk, 2011). There have been calls to drop the label “modern slavery” altogether, in part because the term is used to cover a wide range of practices that cannot be cohesively treated as a single issue (O’Connell Davidson, 2015) and that the focus on the extreme forms of abuse has engendered the view of exploitation as something exceptional (Chuang, 2014). Research has also raised questions about the design and effectiveness of the relevant legal instruments to tackle modern slavery (Balch, 2019; Mantouvalou, 2018; Nolan & Bott, 2018).

Political scientists have focused on understanding the complex economic and political dynamics that induce labor exploitation in global production networks (Barrientos et al., 2013) and questioned the success of voluntary private labor governance (LeBaron & Lister, 2021). They have also analyzed whether and to what extent the modern slavery agenda supports the state’s conservative agendas and immigration controls (Balch, 2019; O’Connell Davidson, 2017) and undermines the legitimacy of trade unions and worker-driven movements (LeBaron et al., 2021).

History scholarship has analyzed the continuities and disjunctures between traditional and contemporary forms of modern slavery, their business models, and anti-slavery measures. However, scholars have criticized the inaccurate use or misuse of history that glosses over legacies of racism (Beutin, 2017) and dynamics of colonialism and patriarchy (Pliley, 2021).

Finally, scholarly debates also concern attempts to quantify the scale and spread of the phenomenon of modern slavery (Phillips, 2018). Measurement of the incidences of modern slavery has been criticized on methodological grounds, in particular the use of poor-quality and limited data (Landman, 2020).

Regrettably, the business and management literature has engaged little in these debates. As noted by Caruana et al. (2021), this lack of attention reflects a longstanding denial of slavery from accounts of modern management (Cooke, 2003). Only recently have management academics begun to participate in these conversations (Smith & Johns, 2020). Modern slavery as a management topic was popularized by Crane (2013), who conceptually linked macro-institutional conditions to firm-level capabilities that help companies reap the benefits of modern slavery, despite such practice being largely illegal and widely regarded as illegitimate. Seeking to explain the “business of modern slavery” (Phung & Crane, 2018), scholars have shown its continuity from traditional slavery (Crane et al., 2021) and have started to unpack the workings of business models of forced labor (Allain et al., 2013).

Supply chain and operations management scholars were among the first to reflect directly on modern slavery (Gold et al., 2015; New, 2015). They provided early definitions and drew attention to *labor* supply chains, which have been neglected in traditional supply chain theory. Other contributions have focused on novel approaches to remediation (e.g., Benstead et al., 2018), and psychological explanations of how purchasing managers’ understanding of modern slavery affects their management of supply chain risks (Simpson et al., 2021). Marketing and accounting scholars have tended to address modern slavery implicitly, through concepts of ethical consumption (e.g., Ballet et al., 2014), corporate human rights, or due diligence (e.g., Methven O'Brien & Dhanarajan, 2016). In their recent review, Caruana et al. (2021) concluded that business and management research into modern slavery is “underdeveloped”; they point out that most work has oversimplified the conceptualization of modern slavery, conflated it with related phenomena, and lacked an in-depth analysis of its dynamics.

The introduction of legislation around the world requiring certain commercial organizations to report on modern slavery (LeBaron & Rühmkorf, 2017; Sinclair & Nolan, 2020) has driven empirical research on the required disclosure and compliance (e.g., Christ et al., 2019; Flynn, 2020; Monciardini et al., 2021; Rogerson et al., 2020; Schaper & Pollach, 2021). In parallel, research interest in how specific groups of business and non-business actors frame modern slavery has grown. This has led to the study of discourses and narratives that draw from ethical considerations and moral arguments (see Dahan & Gittens, 2010, for an early account).

Using a critical discourse lens, Wray-Bliss and Michelson (2021) examined business responses to the Australian 2017 Parliamentary Inquiry preceding the enactment of that country’s MSA. Their analysis reveals that the discursive constructions of “freedom” woven through these submissions sought to achieve minimal legislative burdens (p.2). Embedded in liberal and neoliberal ethics privileging the freedom of the propertied over that of the property-less, the authors show that business’s framing echoes historical business responses toward abolitionist movements in the eighteenth and nineteenth centuries. Using the same Australian empirical context, Christ and Burritt (2018) study the submissions from a broad range of stakeholder groups. Although not grounded in a discursive approach, the authors demonstrate that stakeholders frame differently what “appropriate” business responses to modern slavery should be like. The findings expose a strong stakeholder consensus supporting the introduction of the Act—framed as the means to render modern slavery transparent. Meanwhile, businesses framing emphasizes the value of self-regulation and voluntary codes of conduct in addressing instances of modern slavery.

Narrowing the focus to anti-slavery activists, Islam and Van Staden (2021) capture how their narratives reflect their experience and perceptions on whether the UK MSA has attained normativity, that is, the point at which regulation is regarded as ethically desirable and can exert sufficient moral power to control business behavior. While the study shows anti-slavery activists’ skepticism in the Act’s ability to eliminate slavery, it also reveals how the frame promoted by these actors exposes the lack of corporate transparency as well as the failure to assume responsibility to safeguard workers within global supply chains. Finally, Meehan and Pinnington (2021) draw attention to framing ambiguity in the “transparency in supply chain” (TISC) statements of key suppliers to the UK government. They show that firms use framing ambiguity as a highly strategic mechanism to minimize accountability, delay responses to tackle modern slavery within supply chains, and to distance themselves from collaborative action.

In sum, this research has contributed to our understanding of how discourse and narratives are constructed by specific groups of actors to shape the debate of modern slavery to their own advantage. Yet, only a static account of modern slavery frames has been provided, and there is no dynamic understanding of how these actors’ frames evolve and eventually contribute to or stall the emergence of a common framework of action. Moreover, despite calls to investigate modern slavery holistically (New, 2015) and to consider the interactions of the myriad actors involved in the development of solutions (Van Buren et al., 2021), to date, albeit with a few exceptions (Christ & Burritt, 2018; Dahan & Gittens, 2010; Vestergaard & Uldam, 2021), most research maintains the focus on businesses. In contrast, our work draws attention to a variety of business and non-business actors that through their framing interactions influence the structuration of the field.

### Framing Theory: The Interactive Perspective

Framing is a term often used outside of a formal scholarly discourse. In management and organization theory, its use has become widespread in various streams of scholarship (for a review see Cornelissen & Werner, 2014). In the BE literature, “framing” has been a central cognitive and psychological construct in models of individual and organizational ethical decision (MacLean, 2008; Palazzo et al., 2012; Schwartz, 2016; Shoemaker & Russo, 2001; Sonenshein, 2007). Bringing the sociological understanding of framing to BE scholarship, Dahan and Gittens (2010) elucidate the conceptual and methodological power of this lens for understanding the emergence and contestation of ethical public issues, such as slavery, where multiple meanings coexist and possibly clash. According to Goffman (1974), frames are “schemata of interpretation” (p. 21) that guide actors’ perceptions and interpretations of social reality. Framing involves selection and salience. It operates by focusing attention on an issue, selecting and highlighting some features of the issue while omitting others (Entman, 1993, p.53).

In this paper, we adopt an interactional approach to framing (e.g., Gray et al., 2015) which focuses on how meanings—i.e., frames—are continually negotiated among actors. A growing scholarship using this perspective has elucidated the framing dynamics of various ethical issues^1^ pertaining to the environment and social equity, such as fracking (Nyberg et al., 2020), carbon emissions (Litrico & David, 2017), climate change (Ansari et al., 2013), and humanitarian crises (Reinecke & Ansari, 2016). This perspective is appropriate for our research purposes for three reasons.

First, as with most work drawing on discursive approaches, framing adopts a socially constructivist ontology (Berger & Luckmann, 1966). Framing is seen as the on-going interpretative social process of reality construction (Litrico & David, 2017) through which issues are given meaning (Cornelissen & Werner, 2014). This offers a distinctive ontology to the treatment of ethical issues in typical BE literature (Dahan & Gittens, 2010). Modern slavery is thus not taken as a natural, real or fixed phenomenon but, rather, is understood through actors’ social construction of its meaning (Caruana, 2018). Methodologically, a social constructivist ontology supports an “emic” perspective and a neutral approach that more accurately captures meanings of modern slavery in use by actors (Purdy et al., 2019).

Second, the analysis of framing is a powerful way to connect the processes of meaning making at the meso-level (collective framing of field actors) with the emergence of structure at the field level. This contrasts with the single-actor focus of models of ethical decision-making (Dahan & Gittens, 2010) and is different from the deterministic view in institutional theory depicting field structuration as a top-down process in which diffused templates of meaning are imposed on actors (Gray et al., 2015).

Finally, due to its process-oriented view and focus on political action (e.g., Kaplan, 2008), the interactive framing perspective is better suited than discursive and rhetorical approaches to capturing the negotiation of meaning making (Leibel et al., 2017; Purdy et al., 2019).

Tracing the origin of actors’ frames is beyond the scope of this paper but we acknowledge that frames may be derived from, but not entirely explained by, broad societal logics (e.g., state, market, community, corporation, profession, family and religion) (Purdy et al., 2019) and ideologies^2^ (Creed et al., 2002; Steinberg, 1998). Frames are configurations of building blocks from actors’ menus of cultural repertoires (Kaplan, 2008; Meyer & Höllerer, 2010) and past experience (Cornelissen & Werner, 2014) that influence how actors interpret an issue and subsequently what solutions they propose. Frames do not exist a priori, ready to be invoked, but “involve active struggles and negotiations over meaning before they can solidify and become institutionalized” (Gray et al., 2015 p. 116).

## Conceptual Framework: Dynamics of the Ethical Framing of Modern Slavery

To elaborate our conceptual understanding of the dynamics of the ethical framing of modern slavery, we adopt an issue-field perspective (Hoffman, 1999) and integrate insights from the literature on the discursive construction of moral legitimacy (Palazzo & Scherer, 2006; Scherer et al., 2013).

We draw from a relational notion of fields^3^ (Wooten & Hoffman, 2017) and use Hoffmann’s (1999) definition that a field is a non-physical space formed around an issue that becomes important to the interests and objectives of a specific collective of organizations or actors. “Organizational fields” are thus seen as political arenas where “multiple field constituents compete over the framing of issues and the form of institutions that will guide organizational behavior” (p. 352). The field is thus constituted by specific institutions that lie at the center of an issue-field (regulative, normative and cognitive pillars of institutions) and the individual populations (or classes of constituencies) that inhabit the field (Hoffman, 1999; Zietsma et al., 2017), such as businesses or other organizations that offer similar products or services. Institutions influence organizational behavior. For example, in the present context, regulation (e.g., the UK MSA) guides the interpretation of and behavioral responses to modern slavery; government actors’ framing, particularly during the legislative process, structures how modern slavery is perceived and what actions are developed (Fligstein & Brantley, 1992).

Since modern slavery is a socially constructed ethical issue, there are no objective criteria by which to judge a proposed solution as right or wrong. Each group of field actors with specific interests draws from different moral schemes (Reinecke et al., 2017) and constructs differently the causes of the issue and what constitutes moral solution (Dahan & Gittens, 2010; Reinecke & Ansari, 2016). This moral multiplexity (Reinecke et al., 2017) may create contention. Building on the Habermasian approach to deliberative democracy, Scherer and colleagues argue that moral legitimacy is socially and argumentatively constructed through a communicative process involving dialog between an organization and its audiences (Palazzo & Scherer, 2006; Scherer et al., 2013). Dialog, in turn, prompts actors to be reflective about their moral assumptions, and engages actors in acts of public justification (Reinecke et al., 2017). We thus expect that field actors construct modern slavery frames and deliberate on their moral legitimacy.

### Framing Functions and their Moral Justification

Scholars have noted that frames serve three functions: to punctuate (define what a given problem is), to elaborate (attribute responsibility for the issues i.e., diagnosis and prescribe potential solutions i.e., prognosis) and to motivate (provide conceptual signposts that guide action). In pursuing these framing functions, field actors seek to mobilize both consensus and action (Benford & Snow, 2000). In the case of modern slavery, these framing functions will ostensibly be morally charged (Dahan & Gittens, 2010; Entman, 1993). Field actors will call upon moral values, judgements and principles to convince their target audience. For example, Wray-Bliss and Michelson (2021) found that businesses’ diagnosis of modern slavery involved the construction of businesses as the primary agentic moral subject, which had to bear constrictions of their limited responsibility, and the prognosis of a minimalist regulatory burden. These frames were underscored by the concept of “neoliberal freedom” as a key moral principle of contemporary society. However, research also shows that actors may be reluctant to establish the moral status of their framing as a strategy to legitimize their frames. This is achieved through a process of amoralization (Crane, 2000), by which the ethical issue at hand is rendered amoral. Recently, Vestergaard and Uldam (2021) showed that in their diagnosis, citizens avoided ascribing moral responsibility for modern slavery to companies, civil society or the political system. Relatedly, to establish the moral legitimacy of a controversial issue, actors may instrumentally shift the focus to the moral value of a different issue (Reuber & Morgan-Thomas, 2019).

The framing of ethical issues is not linear (Ansari et al., 2013). To challenge the prevailing order and mobilize action in favor of their own frames and interests, actors engage in framing contests (Kaplan, 2008). When the environment provides political opportunities for action, actors engage in two framing practices: legitimacy claims and frame realignment (Benford & Snow, 2000), also referred as frame shifts (Reinecke & Ansari, 2016). Legitimacy claims are made as actors attempt to bolster their own frames and rebut or challenge the frames of other groups. Frame realignment can have one of four forms: *bridging* (i.e., linking two or more related but unconnected frames); *amplifying* (i.e., invigorating specific elements); *extending* (i.e., incorporating new issues) or *transforming* (i.e., changing old understandings and creating new ones) (Snow et al., 1986).

These two framing practices, legitimacy claims and frame realignment, define what is at play and thus are a means of transforming actors’ interests (Kaplan, 2008). If they are effective, one particular frame will come to prevail and guide actors’ behavior. Subdued and divergent frames will immobilize action. However, actors do not always or automatically interact in their framing practices. Some field actors may be locked in their own frame. This phenomenon, labeled rigid framing (Palazzo et al., 2012), can result in “ethical blindness.”

### Framing Dynamics and Field Settlement

Field settlement occurs when field actors agree on a common frame to guide field-level activities to address the issue at hand (Furnari, 2018; Litrico & David, 2017). Recognizing that settlement can have a diverse field impact, Feront and Bertels (2019) conceptually distinguish three types: rhetorical (where a consensus among some field actors does not lead to new patterns of action); incremental (field actors’ experiment with new practices and policies) and disruptive (substantial changes to field membership rules and/or standards).

Research has traced the evolution of actors’ framing of ethical issues and outlined the antecedents to the emergence of field settlements (Ansari et al., 2013; Feront & Bertels, 2019; Litrico & David, 2017). Field settlements may organically emerge from repeated interactions (Ansari et al., 2013; Meyer & Höllerer, 2010), successive periods of framing contests (Kaplan, 2008), and shifts (Reinecke & Ansari, 2016) that give way to broadly supported compromise or even explicit consensus between field actors (Fligstein & McAdam, 2011). Nevertheless, settlements may occur without a consensus (Cornelissen & Werner, 2014) as long as there is a minimum level of agreement on a joint commitment to the need for action (Ansari et al., 2013); in this circumstance there may be an “optimal frame plurality,” where actors tolerate conflicting frames (Gray et al., 2015; Klitsie et al., 2018).

The strategic efforts of dexterous and dominant field actors who integrate disparate frames into their own framing, or construct more abstract frames that span structurally disconnected frames (Fligstein & McAdam, 2011) are antecedents to field settlement. Settlement can also occur when those actors directly linked to the issue shift their frame from one of denial to one that integrates the issue into their core operations (Litrico & David, 2017). Finally, a settlement may be deferred if frames do not resonate enough to mobilize action or if they remain divergent (Kaplan, 2008).

The emergence of field settlement is not only the result of framing dynamics but is also contingent on the field structure (Furnari, 2018) and power relations that authorize certain actors and perspectives and neglect or exclude others (Meyer & Höllerer, 2010, p. 1251). Centralized fields are characterized by the presence of dominant actors or “elites” possessing legitimate authority (Meyer et al., 1987), while fragmented fields are distinguished by multiple and uncoordinated constituents. Based on this distinction, Furnari (2018) suggest that issue frames identifying an elite as responsible are more likely to trigger a field settlement than frames identifying abstract entities as responsible. Nevertheless, by putting pressure directly on elites, peripheral actors (those disadvantaged by current institutional arrangements) may catalyze critical mass support for their frame and push for negotiation on a new possible framework of action. However, if these elites adopt “rigid framing” (see above) and challengers’ frames are not able to assign responsibility to dominant actors (Furnari, 2018), the elites’ framing risks becoming institutionalized over time (and may even come to be seen as objective truth) and reinforce the status quo.

## Methodology

### Research Design

We have conducted an in-depth, longitudinal, revelatory case study (Yin, 2014) of the framing of modern slavery in the UK construction sector. We started this research in 2015^4^ as part of a wider project to understand how experts in supply management and procurement in construction and other industries were making sense of the prospect of the reporting requirements of section 54 of the UK MSA. In 2014, in the context of upcoming international events such as the 2016 Summer Olympics in Brazil and the 2022 FIFA World Cup in Qatar, the construction industry had been singled out by international unions and NGOs as a risky sector for worker exploitation. Global media started covering stories of abuses by high-profile construction companies. Because of the pressure and international scrutiny, modern slavery concerns surfaced earlier and developed with more intensity in construction than had been the case in other sectors. In 2015, we thus had prima facie evidence that an issue-field was emerging, triggered by the framing of international unions, NGOs, and the media. The framing of modern slavery in construction thus met the criteria for a “revelatory case,” one involving an exemplary phenomenon previously unexplored which enabled us to collect data in real time and analyze the framing dynamics as they were unfolding.

We did not start with a focus on field settlement, but we suspected that the emergence of a common framework of action to tackle modern slavery in the UK construction sector would be resisted due to a legacy of unethical endemic practices (Shah & Alotaibi, 2018) and the sector’s centralized structure (Furnari, 2018). As we were collecting data, the industry started to be heavily criticized for its sluggishness in solving the problem and for its weak compliance with section 54 of the MSA (CORE, 2017; Ergon Associates, 2018; TISC, 2019). This prompted us to narrow our research to the dynamics of framing over time linked to the potential absence of field settlement.

### Data Collection

Our data consists of both primary data and extensive secondary materials (Table 1). Our primary data were derived from naturalistic observations and interviews. Naturalistic observations were made on 12 “field-configuring” events (Anand & Jones, 2008) held between 2016 and 2019—practitioner-oriented construction industry conferences, annual symposiums, initiative launches, and workshops, at which modern slavery or labor exploitation more broadly was a main or featured topic. These events offered the opportunity to capture framing in situ (Purdy et al., 2019) and understand the context in which framing unfolded (i.e., the dialogical nature of framing)—thus facilitating our understanding of which, and how, actors frame issues in a particular way and the interactions among them (Leibel et al., 2017).

**Table 1:** Data sources

Data source	Description of data	Items
Interviews	Fifteen semi-structured interviews with: Sustainable Procurement Manager at a Facilities Management company; Sustainable Supply Chain Manager at Infrastructure group; General Counsel at a construction and property services company; Supply Chain Manager at property support business; Sustainability Manager Construction company; Head of Sustainability at Construction Materials Supplier; Sustainable Procurement Consultant, Business and Human Rights Consultant; Former representative at the IASC office; Policy and Public Affairs Manager of Professional Association Body; Programme lead at initiative to tackle modern slavery; NGO Expert; NGO representative; Lead at Anti-slavery initiative; Former Business journalist	15
Naturalistic observations	Cross-checked field notes (in pages) of field-configuring events including: 2016 Modern Slavery and Ethical Labour in Construction Leadership Symposium; 2017 Modern Slavery and Ethical Labour in Construction Leadership Symposium; 2017 Action Program for Responsible and Ethical Sourcing Annual Conference: Risk & Responsibility: The Evolution of Supply Chain Data and Business Culture; 2018 Action Program for Responsible and Ethical Sourcing Annual Conference: If not now, then when? Responsible Sourcing and Procurement for Infrastructure Projects; 2018 Modern Slavery and Ethical Labour in Construction Leadership Symposium; 2019 Responsible and Ethical Leadership in Global Construction Supply Chains Conference	60
Documentary evidence	Media articles published between 2014 and 2019. Sources included: *The Times, The Guardian, The Independent, The Financial Times, The Sunday Herald, The National (Scotland), Lancashire Telegraph, The Yorkshire Post, The Journal Newcastle, The Irish, The Independent, Plymouth Herald, London Evening Standard, Building Magazine, Labour Research Magazine, Building, Construction News, Building Design, The Lawyer, Thompson Reuters News, Newstex Blog, ENP Newswires, CNN, Source Wire, Mondaq Business Briefing, Financial Wire*	106
	Newsletters and reports from NGOs, professional bodies, industry associations, government authorities, the UK IASC, UK Home Office, management consultancies, accounting firms, and law firms	100

We upheld high standards of research ethics when conducting observations and followed recommendations to deal with emerging challenges (Bryman & Bell, 2011). The researchers attending the events informed the organizers and revealed their identity and the research purpose of the observations. Attendance at these events generated informal data in the form of research field notes with no information that would allow participants or even their affiliations (we used broad categories such as “representative of NGO”) to be identified. The notes mainly captured our key impressions of framing in that event. Video recordings and presentation slides for six of the 12 events were publicly accessible on websites and YouTube. We used this material to complement and cross-check our field notes and to extract verbatim from the contributions of speakers and panelists and thus remain truthful to the actors’ frames and avoid memory losses or bias in our notes. These data were anonymized by removing identifying information.

The cross-checked field notes^5^ were added to the dataset for subsequent analysis. Our own input into the data cannot be ignored. Initially, our observations were unobtrusive and interactions with field actors at these events were limited, but as our attendance became regular, organizers became interested in our project, which led to collaborative relationships that included presenting our findings in one event in 2019.

We conducted 15 semi-structured interviews with UK construction field actors. Our sampling size and selection approach were purposive (similar to Islam & Van Standen, 2021). This meant that the strategy of data collection was driven by reaching data saturation (the point at which subsequent interviews do not provide any additional insights). As per our research interests and theoretical underpinnings, our aim was to obtain a broad representation of participants from the different subgroups of field actors (Table S1 in the Supplementary Appendices), not only businesses. One of the authors, who had established contacts working on modern slavery in a professional role, facilitated introductions to four practitioners. To avoid accessing the views of a particular network of the field, we did not follow a snowballing approach. Rather, we used the attendance at the field-configuring events (above) to purposefully approach additional interviewees based on their subgroup membership. Seven interviewees were representatives of construction businesses, a dominant group in the field, all of which were subject to section 54 of the MSA. Since we used multiple data sources (including the naturalistic observations and extensive secondary data) and the interviews per se were not intended to capture interactive framings, we are confident that the sample size is sufficient for our analysis. Lasting an hour on average, the interviews were intended to elicit perceptions of how the framing of modern slavery and industry actions were emerging and evolving, and to confirm our understanding of the frames identified. The interviews were recorded and transcribed for subsequent analysis. We requested participants’ feedback on preliminary findings in order to ensure the accuracy of our interpretations.

Our collection of secondary data involved building a database of UK major daily newspaper articles, newswires and press releases, web-based publications, and the industry trade press. This enabled us to place our case in a broader context and track the evolution of frames over time. Using keywords such as “modern slavery,” “forced labor,” “worker/labor exploitation,” and “construction,” we collected a total of 106 articles published between 2014 and 2019 from the LexisNexis database. To capture frames that had failed to penetrate the mass media, archival data included different sets of texts reflecting subpopulations’ framing within the field (Leibel et al., 2017). For example, unions’ framing was more prominently featured in outlets such as *Labour Research UK*, a specialist magazine for union representatives that provides regular updates on workers’ rights and employment law, rather than in major daily newspapers. Thus, we included media outlets such as local newspapers, industry newswires, construction magazines, and blogs. Additionally, we collected 100 documents, including newsletters and reports from various field actors.

Our strategy of combining different sources of qualitative data enabled us to triangulate data, validate insights, and increase the study’s internal validity.

### Data Coding and Analysis

We adopted a semi-inductive approach to data analysis in three inter-connected stages and drew on established methodologies for the analysis of ethical issue frames (e.g., Dahan & Gittens, 2010) and framing dynamics (e.g., Ansari et al., 2013; Kaplan, 2008; Litrico & David, 2017).

#### Stage 1: Organizing Data Temporally and Identifying Key Field Actors

To condense data and organize data chronologically, we used “temporal bracketing” (Langley, 1999). Content analysis of media articles was used to identify and characterize events that might drive the evolution of framings. We developed an event history database, summarized in Table S2 (Supplementary Appendices). The event data showed turning points that enabled us to distinguish three periods.

As per our conceptualization of the issue-field and recommendations of how to analytically detect it (Hoffmann, 1999), we paid attention to the actors that engaged in the modern slavery debate in construction. Membership to the field was thus not externally imposed but emerged from the data (i.e., actors’ framing patterns and interactions). For each article in our data set, we coded whom or what the article was written about, the topic the article covered, and other actors mentioned; anyone whose view on modern slavery was directly quoted or paraphrased was considered a focal actor. If no particular speaker was mentioned in an article, we ascribed statements to the journalist.

#### Stage 2: Identifying Frames, their Functions and Moral Justifications

The cross-checked observational data, media database, and additional documents were analyzed semi-inductively. We used a “signature matrix” (Creed et al., 2002, p. 40) to capture the building blocks of a frame (metaphors, exemplars, catchphrases, depictions, visual elements, roots, consequences, and appeals to principles). Each text was coded by the first author according to how the actor(s) framed modern slavery. Second and third authors were not directly involved in the coding but settled potential discrepancies. To identify frames, we followed Benford and Snow’s (2000) three categories of framing functions (punctuation, elaboration, or motivation) and discerned the moral justifications provided. We manually coded the dataset using a master spreadsheet. Accordingly, we defined separate framings if the punctuation, elaboration, or motivation function differed. We then refined these codes as we engaged with the data, paying attention to repeated patterns and who was an advocate or challenger of a certain frame. We “stacked” each frame in a matrix for systematic comparison. This process led us to identify and define five distinct frames: (1) human rights issue, (2) moral issue, (3) management issue, (4) social justice issue, and (4) decent work issue. We then asked our interviewees to establish whether they considered this mapping of frames to be accurate. Table S3 (Supplementary Appendix) provides illustrative quotes.

#### Stage 3: Identifying Field Realignments and Settlement

We qualitatively tracked the patterns of interaction and the use of frames over time. We sought evidence on whether field actors’ interpretations of modern slavery diverged from the common meaning of each of the frames initially identified, and if so how. We recorded subtle changes during our observations in field-configuring events, which we followed up, seeking evidence of any suspected pattern. For example, “hidden” and semantically related words were used in the early stages of framing and prominently used by some frame sponsors from 2018. Similar patterns were observed for the use of words such as “human beings” and “victims” from 2019. Frequency word queries confirmed our observations.

We identified three realignments and coded their features by drawing on the literature. Two realignments corresponded to subtle variations in the punctuation and elaboration functions the human rights issue and the “management issue” frame. The third realignment corresponded to a frame break (Goffman, 1974), when a new frame, namely “decent work”, sponsored by actors originally attached to a different frame, emerged to challenge and revise the existing field frames. Using the event history database produced in stage 1, we assessed the conditions underlying these realignments We then assessed whether actors’ frames changed in relation to any of the events. This process focused on connecting macro-processes of contention in the public sphere with the meso-processes of framing in the field (Steinberg, 1998). We identified the type of process underpinning each realignment (e.g., Snow et al., 1986): the two shifts were underpinned by *amplification* and *extension* processes respectively and the frame break was underpinned by a *bridging* process.

To analyze field settlement, we examined the reactions of groups of actors and developed related coding. This revealed the emergence of various multi-stakeholder initiatives (MSIs) seeking to address modern slavery in the industry. Using existing categorizations of industry MSIs on human rights (e.g., Baumann-Pauly et al., 2017), we analyzed their purpose. In line with prior studies of field settlements (e.g., Feront & Bertels, 2019; Litrico & David, 2017), we categorized the field impacts of MSIs using codes such as “business-as-usual,” “discursive alterations to policies,” and “inconsequential changes.” This enabled us to contrast these findings with prior work distinguishing “rhetorical,” “incremental,” and “disruptive” field settlements (Feront & Bertels, 2019).

### Research Context: The UK Construction Sector

The construction sector is a crucial part of the UK economy, representing 6% of its total economic output, employing 2.34 million people, and involving 17% of its businesses (Rhodes, 2019). Construction is especially prone to exploitation (UK Home Office, 2019) because of the high proportion of low-skilled and migrant workers (CITB, 2017); the high level of subcontracting (SMEs comprise 99.9% of construction contracting businesses while large companies are mainly involved in project management or materials supply) and the lack of visibility below the first and second tiers of the supply chains (Department for Business Innovation & Skills, 2013); project-based and short-term relationships with contractors; over-reliance on labor agencies; low margins, with some of the UK’s top ten contractors making less than 1% profit (Financial Times, 2018); and several endemic industry practices, such as lowest-cost tendering, project discounting, retentions, and late or non-payment (CIOB, 2016). These factors have come into the limelight because of recent scandals such as the liquidation of mega-contractor Carillion in 2018, price cartels, and industry-wide union busting (BBC, 2019; Harper, 2018).

Albeit a fragmented and loosely coupled sector operationally (Dubois & Gadde, 2002), UK construction can be considered a centralized field (Furnari, 2018). It is characterized by the presence of a relatively small number of “elite” organizations that exercise purchasing and regulatory power and control by constructing shared norms. These dominant organizations include large client and project management companies, public procurement bodies, trade and professional associations, certification bodies, industry skills and standards bodies, and industry knowledge initiatives (see Table S1).

## Findings: Dynamics of Modern Slavery Framing

Our empirical findings are structured into three periods: emergence, intensification, and disenchantment. For each period, we organize and present the actors’ group-level frames of modern slavery and frame realignments. Each frame and its trajectory are summarized in Table 2. We integrate authentic voices into the presentation of our findings in order to illustrate their views and reduce the scope for bias.

**Table 2:** Framing of modern slavery

	Human rights issue	Moral issue	Management issue		Social justice issue	Decent work issue
Field actors advocating the frame	UK politicians: Prime Minister, cabinet members, MPs, Home Office and foreign affairs officials	Representatives of professional association bodies, MSIs, think tanks, industry knowledge initiatives, and a cross-bench peer	Representatives of large businesses, lawyers, consultants, a few cabinet ministers, representatives from some NGOs, and industry knowledge initiatives		Campaigners, activists, Labor party leaders, NGOs representatives, watchdogs, government agencies, the IASC, selected consultancies, unions, and a cross-bench peer	Representatives of professional association bodies, some industry knowledge initiatives, some business “champions”
Punctuation function	Defines modern slavery a crime	Defines modern slavery as the result of an immoral professional decision	Defines modern slavery as a management issue		Rejects modern slavery framing and focuses on labor exploitation	Shifts attention to the issue of decent work
Elaboration function						
Diagnostic framing	Individual morality of traffickers	UK shortage of labor and desperate managers	Global supply chains		Combination of neoliberal economic and social system inducing the demand for cheap labor and decline in worker’s protection	The industry (business models, culture, and professionals)
Prognostic framing	Structures to prosecute and disrupt criminals	More severe civil and criminal penalties and prosecution of professionals	Fatalists: Skeptical about solutions	Optimists: Corporate-driven solutions compatible with MSA	No solutions outlined	Verticalization and decommodification of workers’ labor
Vision of solutions	Long-term	Moderately urgent	No urgency	Long-term		Collaborative
Motivation function	Action mobilization: Calls to the UK to continue as leader in abolishing slavery	Consensus mobilization: Calls for leadership and reform in the industry	Absence of action mobilization	Action mobilization: Business case to tackle modern slavey	Action mobilization: Calls to disrupt the system, reconfigure relations and confront corporate power	Consensus mobilization: Calls for an introspective assessment of industry practices
Moral justifications	Legacy of British abolitionism and UK’s moral sense of ‘goodness’	Ethical case aligns with interest in sector’s performance and long-term growth	Amoralization of modern slavery Maximization of profits aligned with interest of modern slavery victims		Normative superiority on worker’s rights over efficiency gains	Respecting workers’ rights and ensuring decent work as moral priorities
Frame trajectory	Originates in ‘emergence’ period, shifts into hidden crime frame in ‘intensification’ period, and persists throughout ‘disenchantment’ period	Originates and dissipates in ‘emergence’ period	Originates in ‘intensification’ period and shifts into human moral obligation in ‘disenchantment’ period		Originates and dissipates in ‘intensification’ period	Originates and persists throughout ‘disenchantment’ period

### Period I: Emergence

In 2014, international trade unions challenged the organizers, host governments, and multi-national construction companies over the treatment of workers for the construction of facilities, infrastructure, and stadiums for the 2016 Summer Olympics and the 2022 FIFA World Cup. The publication of the report *The Case Against Qatar* by the International Trade Union Confederation in March 2014 conceptually marks the formation of the issue-field (Hoffman, 1999). A subsequent report by Amnesty International, *Promising Little, Delivering Less*, on Qatar’s proposed reforms to tackle modern slavery intensified global media attention on the issue.

These events initiated scrutiny on the UK construction industry and triggered the framing and subsequent interactions among actors. The first framing period thus covers the legislative process in the UK Parliament that led to the enactment of the MSA in October 2015.

#### Frame 1: Human Rights Issue

This frame originated in the media coverage of exploitation and abuse in large-scale construction projects in Qatar and the UK. Advocates of this frame, including the UK Prime Minister, cabinet members, Members of Parliament (MPs) and Home Office (HO) and foreign affairs officials, define modern slavery as a human rights violation. This frame dominated the field in the run-up to the 2015 enactment of the MSA as hearings took place in the House of Commons. Advocates of this frame wanted to garner political support for the government policy to tackle modern slavery. Their rhetoric projects anger over and moral disapproval of these abuses, referring to modern slavery using terms such as “global disgrace,” “shameful practice of trading of human beings,” and “rotten, grotesque and evil practice.” In their diagnosis, frame sponsors decidedly identify those culpable as the traffickers. The frame thus locates the source of the problem in the morals of “criminals” and “aggressors” and those who tolerate such practices. In doing so, this frame conflates modern slavery into a dichotomy between the perpetrators and the victims. The latter, often described as “naïve” or “tricked,” have all agency removed from them in this frame. Advocates occasionally accused labor agencies of complicity in the crimes but rarely blamed the businesses hiring labor from them. On the contrary, many politicians praised businesses’ efforts:

“UK businesses are leading the way on work to create a human rights benchmark so companies around the country and international firms could be compared on their record.” (Representative of the Department for Business, Innovation and Skills, quoted by the Press Association, December 16, 2014)

In summer 2014, while the Modern Slavery Bill was being discussed, including the proposed legislative options to address forced labor (LeBaron & Rühmkorf, 2017), frame proponents argued that the MSA would become model legislation, particularly in its requirements for transparency, and would show the “way forward to reduce the risk of modern slavery” (MP, 2014). At this stage, frame advocates did not frame reporting requirements as the solution, but misleadingly equated them to a public confirmation that supply chains are slave-free.

“Its new requirement [the MSA] for companies to publicly report on their supply chain's freedom from the employment of slave labour will ramp up the reputational damage and possibly the legal damage that such an offence would cause.” (MP, quoted in *Building Magazine*, December 12, 2014)

These advocates saw the lower end of the labor supply chain as requiring regulation and the construction contractors, at the upper end, were implicitly free of blame for any infringement of human rights. Furthermore, proponents portray the corporation and its supply chains as being subjected to the “crime”.

In their prognosis, frame proponents converged in solutions that consistently targeted the culpable (i.e., the traffickers) and aligned with one of the four components of the UK government’s modern slavery strategy, namely the “pursue pillar” (i.e., prosecuting and disrupting individuals and groups responsible for modern slavery). The HO uses the same principles in its anti-terrorism strategy. Proponents thus believed that the government should “strengthen the infrastructure” to “inspect, catch and prosecute the criminals.” To mobilize support and establish the moral legitimacy of their frame, sponsors invoked British abolitionism and constructed a celebratory tale of the “historical” and “leading” role of the UK. Advocates frequently draw comparisons between the Modern Slavery Bill and the 1807 Act for the Abolition of the Slave Trade in the British Empire and emphasized the country’s moral sense of goodness in “saving” modern slaves. They argue that the UK should take a stand to show the world that it will not profit from exploitation. Advocates believed that although progress had been made, there was much more to do. The time frames suggested (between 20 and 30 years) signaled their long-term vision.

#### Frame 2: Moral Issue

A second frame that emerged before the MSA’s enactment was the moral issue frame, which coexisted with the human rights frame. This frame was distinctively constructed by representatives of professional associations, think tanks and industry knowledge initiatives, together with a cross-bench peer who campaigned for legislation in supply chains in the garment industry alongside the creation of the MSA. This group defined modern slavery as the result of an immoral professional decision. Advocates recognized that construction professionals constantly had an urgent need for labor to meet project deadlines and had to make an ethical choice on whether or not to “cut corners” and “turn a blind eye” to the practices of organizations supplying labor. The diagnosis was the labor shortage in the UK. Advocates often used economic estimates and statistics in this framing of the problem:

“The sector's skills body, the Construction Industry Training Board, says that to cope with rising workloads almost 224,000 new recruits will be needed between 2015 and 2019. To address this, the industry has a simple set of choices: import from a ready supply of foreign talent, invest in training and development of UK citizens, or redesign the construction process.” (Chartered Institute of Building report, 2015)

While frame advocates ultimately placed the blame on the ethical decision-making of professionals, they also perceived that “the right thing to do” for a construction professional is tarnished by a pervasive corporate culture in the construction sector that tolerates unethical conduct. For example, advocates frequently compared modern slavery to the corruption that had tainted the industry’s reputation. Interestingly, this is the only frame in our study in which industry professionals were urged to choose “what is ethical” rather than “what is legal” (implying that modern slavery may occur under legal compliance). The following quote from the CEO of one of the associations for construction professionals illustrates this:

“The industry has a moral duty not to collude in the exploitation of vulnerable people. Clients and principal contractors should take a responsible attitude to exploitation, even if they are not obliged to do so contractually. It’s being done in their name after all.” (*Building Magazine*, August 28, 2015)

To build the moral soundness of their “ethical case to react,” this group ranked the construction sector’s performance, productivity, and long-term growth as high-order principles. They feared that not acting ethically would damage the industry.

Advocates tried to mobilize consensus by calling for “strong leadership” to persuade professionals to adopt more ethical practices. Further, the industry’s inherent culture was seen to be in need of “reforms” to accelerate change, but advocates were vague about what this would entail.

In their prognosis, frame proponents recommended more severe civil and criminal penalties for the employment (even if unknowingly) of “illegal workers,” but also emphasized their interest in raising awareness and educating industry professionals through the dissemination of information and open discussion.

### Period II: Intensification

The second framing period starts from the enactment of MSA in October 2015 and ends in September 2018, when the government commissioned an independent review of the working of the MSA. A key event in this period was the first reading of the Modern Slavery (Transparency in Supply Chains) Bill, introduced in the House of Lords in July 2017, which sought to amend section 54. During this period, framing intensified and field-configuring events burgeoned.

#### Frame 3: Management Issue

After the MSA became law in 2015, the management issue frame emerged and soon became frequently used. It remained active until the end of period III. Frame proponents were the managers of large businesses,^6^ lawyers, consultants, a handful of cabinet ministers, and representatives of some NGOs and MSIs. In this frame, modern slavery is seen as a management issue, whereby some firms profit from treating workers as “the lowest commodities.” Advocates expressed unease that some companies had been able and willing to take advantage of the conditions that allow slavery to flourish (Crane, 2013); indeed, in this frame slavery was seen as “big business.”

In their prognosis, proponents did not assign culpability to nameable actors but instead identified modern slavery as the inevitable result of globalized supply chains. A strategy of amoralization (Crane, 2000) was thus used by advocates to deny any corporate responsibility for modern slavery. Their framing precluded any contribution of *their* business to modern slavery.

Although agreed on the prognosis, proponents were divided into two groups in their diagnosis, the “fatalists” and “optimists.” Fatalists were skeptical about whether the problem of modern slavery could actually be fixed, because it was “too complex,” “daunting,” and “immense,” and has “too many unknowns.” They argued that the structure of the global economy meant that one single actor could not possibly end the exploitation in complex supply chains. This group was generally unconcerned with mobilizing action and repeated phrases such as “We have to wait and see,” thereby detaching themselves from any moral agency and avoiding urgency. This group’s interests were very narrow, and mainly concerned compliance with the reporting requirements and minimizing the risk of being implicated in modern slavery.

Unsurprisingly, the majority of optimists used business case rhetoric to mobilize action to tackle modern slavery. They viewed the issue as a business opportunity to create value, innovation, and productivity: “those that invest will see the payoff.” Some linked the identification of the risks of involvement in modern slavery with the bottom line:

“Businesses that identify general areas where the risk of adverse human rights impact is more significant will drive improvement and return on investment.” (Head of Advisory & CSR of a consultancy, field-configuring event, 2017)

Occasionally, proponents used inverse framing of this argument: “if business leaders do not address the issue, profits will be damaged” (Sustainability consultant, 2017). Thus, optimists’ framing conveyed a “win–win” situation similar to that described by Monciardini et al. (2019, p.35) in which businesses’ interests (i.e., maximization of profits) align with those of modern slavery victims. These findings are thus in stark contrast to the “ethical case” championed by the moral issue frame advocates.

Corporate heads of CSR, sustainability, and human rights repeatedly advised peers to deal with modern slavery as a health and safety (H&S) issue:

“You should think and treat modern slavery as a risk in terms of H&S because bringing someone without skills or training to the site has dangerous consequences for the business.” (Human rights manager, field-configuring large construction company, 2016)

Relatedly, they argued that modern slavery is correlated to right-to-work checks in the UK, which shifts attention from the moral issue of exploitation of people to firms’ reputational risk of employing illegal workers. Thus, similar to what was observed in the human rights issue frame, the “management issue” frame places moral worth on the corporation by centering on the impacts on the firm rather than on the impacts on human lives.

As the MSA came into effect, lawyers, management consultants, and some representatives of industry-led initiatives and NGOs were prominently featured in field-configuring events. Their prognosis spanned various corporate-driven solutions “to prevent and tackle modern slavery” and they advised companies to produce an MSA statement even if they had not acted to tackle the issue. Optimists pointed to mapping operations and risk assessments; developing robust recruitment checks and proper background screening of labor agencies; self-assessment tools, audits, and questionnaires for suppliers; and training on “zero-tolerance policies and approaches.” The latter solution aligns with suggestions proposed in the human rights issue frame. For this group, businesses’ goal was to demonstrate that their supply chains were free of slavery:

“We believe slavery has no place in the modern world and we take a zero-tolerance approach. We are committed to ensuring there is no slavery in any part of our business or supply chain, and we are implementing and enforcing effective systems and controls to enforce our approach, extending to our own employees and the thousands more employed in our supply chains, including subcontractors, suppliers, and labor agencies.” (Chief executive of a large family-owned construction and development company, quoted in the London *Evening Standard*, 24 November, 2017)

A minority of consultants championing these initiatives also acknowledged the limitations of these approaches: “Map your risks but do not wait to hear back from a letter you sent to your suppliers in which you asked them whether they have slaves or not… This is not a tick the box exercise” (Sustainable Procurement and Modern Slavery Senior Consultant, 2017).

Optimists underlined that these actions should be incremental—“start with a little,” “focus on your first tiers,” and see this as a “long-term journey.” Fatalists expressed fears of being targeted by the media as companies’ first and second MSA statements were scrutinized in period II. They demanded immunity and protection from reputational risk to enable open reporting about their supply chains and detected cases of modern slavery. We noticed disagreement over the costs and benefits of compliance and non-compliance with reporting requirements. While fatalists considered open and honest reports to be disadvantageous, optimists called for the punishment of non-compliant firms and rewards for compliance, through “blacklisting” and “shame and fame” mechanisms. Other disputes concerned the purpose of MSA statements. Some argued that they should be aspirational, while others asked peers to “keep it real” by reporting verifiable facts for which they could be held accountable.

#### Frame 4: Social Justice Issue

Campaigners and activist groups, members of the Labor Party, representatives of some NGOs, watchdogs, some members of the Gangmasters and Labor Abuse Authority (GLAA), cross-bench peers, the former Independent Anti-Slavery Commissioner (IASC), specialized consultancies, and fringe actors such as unions constructed a frame that we label “social justice.” Its proponents expressed a broad interest in tackling social inequalities and defending civil and labor rights.

This frame developed in parallel to the management issue frame and appeared mainly in print media. Advocates were notably absent from field-configuring events. The social justice frame rejects the “modern slavery” label, arguing that it distracts from other forms of labor exploitation and ignores its structural causes (power imbalances in the economy). The frame encompasses an assessment of the functioning of both the UK and the global economy. Proponents perceived modern slavery as being caused by the “neoliberal economic and social system,” a combination of “the demand for cheap labor” and the decline in workers’ protection, including the deterioration of union representation and the absence of collective bargaining in the UK. The diagnosis thus gives workers’ rights, grounded in principles of human dignity and equality, normative superiority over efficiency gains. Besides the economy, “a broken system” and “the establishment” are other abstract entities blamed for the failure to protect vulnerable sections of society:

“Their general feeling [victims]—and I totally get this—is that the establishment doesn’t really care about them.” (Migration policy and services coordinator of an NGO*,* quoted in the London *Evening Standard*, November 24, 2017)

Using rights-based arguments, they heavily opposed the criminal justice solutions proposed by the human rights issue frame and private initiatives such as CSR. They condemned these approaches for undermining a labor perspective and diverting attention from the core problem:

“This issue is mainly about employment relations, but you cannot start solving this issue with some people sitting in a room looking at a spreadsheet.” (Former IASC, field-configuring event, 2016)

Turning their attention to the human rights issue frame, the sponsors of the social justice frame extensively denounced the Conservative-led government’s approaches to tackling modern slavery. First, they criticized the focus on “catching and prosecuting the traffickers” instead of preventing modern slavery and protecting victims, particularly when inspecting and prosecuting agencies (e.g., the GLAA) were facing significant cuts.

Second, adherents criticized the government’s “paradoxical” response and “hypocritical morality” in devising the MSA while simultaneously criminalizing undocumented workers. Proponents highlighted the conflict of interest in combining immigration enforcement with labor inspection powers:

“The Home Office faces a conflict of interest between its responsibility to identify and protect victims of trafficking and its role in detaining and removing undocumented migrants. The prioritisation of these enforcement responsibilities leads potential victims of trafficking to be detained without careful assessment of their situations.” (human rights campaign group report, November 20, 2017)

Finally, frame proponents contended that the government was protecting the private sector by devising a weak law (the MSA), allowing businesses to get away with “saying too little” and not complying with obligations such as paying workers the minimum wage. They criticized the government’s assumption that cases of modern slavery are businesses’ “unintentional errors.”

Advocates of this frame sought to mobilize action by rallying actors to “disrupt the system” and “reconfigure relations of the wider system,” and “confront corporate power,” elevating workers’ rights over profits, but they were vague on prescribing a concrete set of actions.

#### Frame Shift 1: Recasting Modern Slavery from a Human Rights Issue to a Hidden Crime

By the end of December 2017, two reports^7^ on the UK and police response to modern slavery showed that the HO had an incomplete picture of the crime obscuring the identification and prosecution of individuals and that the police lacked understanding of the nature and scale of the issue. The publication of these highly critical reports appears to have driven a subtle shift in the human rights issue frame, which was now recast in terms of “hidden” crime.

Initial proponents of the human rights issue frame contributed to this shift, which was supported by representatives of some NGOs, MPs, the National Crime Agency, Scotland Yard, the HO, the National Referral Mechanism (NRM) and senior policing figures. Frame proponents drew attention to the concealed nature of modern slavery, frequently using expressions such as “it grows in the dark” and “it’s hidden in plain view”:

“Human trafficking and exploitation prey on the most vulnerable in society. Often hidden in plain sight and in legitimate businesses, these offences are on the increase worldwide.” (Deputy leader of a UK political party, quoted in *Daily Business*, October 18, 2018)

Some continued to draw ahistorical comparisons to transatlantic slavery but the focus was increasingly on the difficulty of detecting the crime nowadays. Frame proponents continued to blame “traffickers” but also now accused them of “infiltrating legitimate businesses.” Similar to the amoralization strategy used by the advocates of the “management issue” frame, proponents continued to minimize businesses’ moral responsibility for the problem by portraying them as victims:

“While it may be unlikely that large companies are directly employing trafficked people, contractors and subcontractors or the agencies supplying labour could find themselves targeted by unscrupulous gangmasters who may be offering a ready supply of labour at knocked down rates.” (HO fact sheet used at a field-configuring event, 2018)

Thus, this shift reinforced the widespread assumption of the absence of business wrongdoing, condemned by social justice frame proponents.

To invigorate the frame, frame advocates engaged in a process of *amplification* (Snow et al., 1986) by building a logical link between the solutions (focus on transparency and identification) and a characteristic of the issue (its hidden nature).

Proponents thus focused on legitimizing the normativity of the MSA and championing the structure established by government and labor agencies, such as policies and mechanisms to help victims come forward (e.g., the NRM and the Modern Slavery Helpline) and platforms for businesses to share information with police intelligence (the GLAA Construction protocol). They also endorsed campaigns to raise awareness and educate the public to “spot the signs” and emphasized the importance of reporting cases to the police. The following quote from an HO representative illustrates the connections between the “hidden” nature of modern slavery and the evidence that government policies were working:

“Our policy is designed to encourage more victims to come forward and ask for help. We welcome increases in the number of referrals as a sign that our efforts to shine a light on modern slavery are working.” (Nexis Lexis, October 10, 2019)

### Period III: Disenchantment

This period starts in September 2018, with the UK government’s commissioning of an independent review of the MSA. It covers the review process, the government-launched consultation, and the government’s response to the independent review recommendations.

Period III also covers the on-going legislative process of the TSC Bill, which had begun in Period II but had been much delayed by the legislative and political demands of Brexit. By the end of the study period in 2019, it had still not progressed to a second reading in the House of Lords.

#### Frame Shift 2: Management Issue Frame Infused with a “Human Moral Obligation”

By the end of 2018, a wave of reports had exposed disappointing compliance with MSA Sect. 54 (e.g., CORE, 2017; Ergon Associates, 2018) and official figures showed a substantial increase in allegations of labor exploitation. Proponents of the management issue frame raised concerns about peers who seemed to be treating disclosure mainly as “paperwork,” with only a minority “walking the talk.” It is in this context that the management issue frame underwent a process of *extension* (Snow et al., 1986) whereby the ideational element of “human moral obligation” was added to the frame to achieve resonance with other audiences. Those proponents, led by a coalition of “best players,” created a narrative calling for businesses to “humanize” their responses to modern slavery by focusing on preventing and mitigating risks to people instead of risks to business. They made appeals to their peers’ moral duty: “We are dealing with humans,” “Never go away from a victim,” “We need to stop seeing workers as expendables or commodities.”

To build broader support for this extended frame, the organizers of field-configuring events sought to establish emotional connectivity (Gray et al., 2015; Reinecke & Ansari, 2016) with the audience. They invited former victims of modern slavery and the producers of theatrical productions on modern slavery as panelists, and showcased films depicting “real stories” of the exploitation of construction workers. Corporate speakers used anecdotes to draw attention to the victims and the corporate responses, as illustrated in the following quote:

“The really good thing to me is that we are starting to talk about victims, because this isn’t about statements, it’s not about pieces of paper and partnerships and logos and all those things that corporate PR people seem to get excited about. This is about going into supply chains, finding, fixing and needing to work with the right partners to take those victims out of the appalling situations they are in and getting back them into society.” (Head of sustainability of a large materials firm at a field-configuring event, 2018)

A significant contributor to this shift was the demystification of the profile of “modern slavery victims.” Management frame proponents as well as other field actors assumed that victims were mainly immigrant and low-skilled workers who had been “trafficked.” As the NRM’s statistics were released, these proponents obtained a more nuanced picture of victim profiles, in which some of these workers had not been forced to move but had in fact exerted their agency and sought greater freedom in coming to the UK:

“Modern slavery victims are vulnerable parts of society, not necessarily unskilled workers, they can be engineers … and highly skilled foreign workers from overseas but also Europeans with a ‘right to work’.” (Representative of MSI to tackle modern slavery in construction, field-configuring event, 2018)

During this period, frame proponents intensified their recommendation that firms place victims at the center of their investigations, program developments, and remedial actions. Corporate actors considered “champions” (i.e., firms with longstanding sustainability reputations) inside and outside the field were featured as exemplary cases. For example, at one field-configuring event, the sustainability marketing director from a large materials firm emphasized that victims were central to the company’s modern slavery response, supported by covert intelligence-gathering in collaboration with an investigative NGO and appropriate remediation programs. Relatedly, examples of collaborations between businesses and charities providing employment for modern slavery victims were promoted. These examples were echoed by facilitators in training delivered to procurement, legal, CSR, and HR professionals. However, some advocates of the management issue frame rejected the suitability of a “victim-led” approach. In parallel, proponents of the social justice frame feared that this would exacerbate workers’ vulnerability, by putting them at risk of deportation when referred to authorities. Some NGOs used expressions such as “you are not expected to deal with suspected victims” and “that’s not the remit of corporations.”

#### Frame Shift 3: Emergence of the Decent Work Issue Frame

During the summer of 2019, the UK government held a public consultation on the transparency, or reporting, provisions of the MSA. In its response to the recommendations of the independent review, the government indicated it would “strengthen” and “future-proof” the MSA. In parallel, there was extensive media coverage of the conviction of members of a Romanian criminal organization trafficking victims into the UK construction sector. We noted that several field actors, originally optimistic about the MSA’s prospects for catalyzing change, started distancing their prognosis frames from the MSA and felt increasingly compelled to react because of the increased scrutiny of the sector. The third shift identified was the emergence of a new frame focused on the issue of “decent work.” Advocates argued that the term “modern slavery” suggested the problem was “atypical,” whereas it was in fact prevalent within the sector.

Proponents of all four of the previously established frames participated in constructing this frame, by bridging elements of the moral and social justice issue frame and promotes the revision of dominant frames (human rights and management issue frames in periods I and II).

In their diagnostic frame, proponents of the decent work frame asserted that the problem lay in the industry itself, and this marked an important departure from previous frames, which all denied corporate moral responsibility, instead assigning responsibility to organized criminal gangs or “global supply chains”. In this new frame, advocates propose to fix the industry from the inside. Frame proponents called for an introspective assessment of the sector, which had “portrayed itself as the victim.” Their diagnosis focused on the sector’s business model, culture, and professionals.

Proponents described the industry as a “house of cards,” with a low-margin business model inducing a “spiral of not getting out of exploitation” i.e., subcontractors are not interested in working conditions and thus indirectly incentivize exploitation in labor supply chains. Advocates blamed an industry culture that was hostile, adversarial, and unwilling to collaborate, which pushed professionals (particularly in procurement) to act on a purely transactional basis. Original supporters of the moral issue frame highlighted the absence of a “license to practice” for construction professionals and lack of strict ethical codes of conduct and accountability mechanisms.

Proponents of the “decent work” frame suggested that the solution (or “prognosis”) was to tackle the “bundle” of underlying conditions that were driving labor exploitation, such as extensive subcontracting, over-reliance on labor agencies, and the pervasiveness of short-term contracts. An innovation of the “decent work” frame was that the solutions were now independent of the MSA’s reporting requirements. Thus, proponents recommended “going back to basics” and a focus on “tangibles,” which broke with the view of slavery as a “hidden practice.” They proposed a focus on verticalization (i.e., reducing the number of links in supply chains) with worker recruitment concentrated inhouse and workers’ labor decommodification by paying “living wages” (rather than minimum wage), looking after workers’ welfare, and reducing short-term contracts:

“We need to increase the proportion of labor directly employed on projects and focus on what is tangible: improve living standards and conditions.” (Sustainability advisor, certification body, field-configuring event, 2019)

Similar to the diagnosis within the social justice frame, this frame diagnosis implicitly placed a moral priority on respecting workers’ rights and ensuring decent working conditions. To mobilize action, proponents envisioned achieving these solutions through collaboration, both intra-organizational (avoiding silos inside firms) and inter-organizational (among peers and other field actors).

Criticisms of the solutions proposed by proponents of the management issue frame were intensified within the new frame. Advocates condemned the use of social audits, zero-tolerance policies, and reliance on advice from consultants and lawyers on the content of MSA statements. They argued that “businesses need to conduct their own forensic work, take ownership of the issue and open up about it” (representative of an industry knowledge association, 2019), even if this meant having “uncomfortable conversations,” which clashes with the demand for “immunity” from proponents of the management issue frame.

### Business-as-Usual but Change in Rhetoric?

We did not find evidence of business actors substantially changing their practices or of new field membership rules and/or standards by the end of 2019, when data collection ceased. Observable changes were limited to the emergence of corporate-driven solutions proposed by advocates of the management issue frame, aligned with existing practices such as CSR. Some NGOs had started to coordinate efforts with businesses, professional bodies, and government agencies to develop voluntary MSI, some of which made rhetorical changes to policies aligned with the “decent work” frame.

We identified seven MSIs: the BRE Ethical Labour Sourcing Standard BES 6002, the Action Programme on Responsible and Ethical Sourcing (APRES) Eight Pathways Model (best practice), the CIOB-Stronger Together Modern Slavery Toolkit, the Construction Coalition Charter, the GLAA Construction Protocol, the Building Responsibly Worker Welfare Principles, and the Supply Chain Sustainability Procurement Guidance.^8^ Some of them specifically address modern slavery while others cover aspects of ethical sourcing and sustainable procurement. These MSIs mainly focus on raising awareness, defining broad principles of worker welfare, raising standards (i.e., moving from “baseline” to “best-in-class” performance), outlining criteria for certification, sharing intelligence, and exchanging best practices. Some provide practical guidance on how to mitigate modern slavery within organizations’ own operations (including subcontractors, suppliers, labor providers, and services) and on which functions to report in annual slavery statements.

Close inspection of these initiatives reveals some developments. The Supply Chain Sustainability Procurement Guidance provides specific recommendations on amending pre-qualification questionnaires to incorporate the MSA, either to select suppliers based on their attitude to modern slavery or to introduce requirements and continuous improvement targets as part of the contract award. However, these guidelines’ key message is that modern slavery is a new issue that must be solved within the usual business model and internalized within existing processes and structures.

The CIOB-Stronger Together Modern Slavery Toolkit resonates with the prognoses of the management issue and decent work frames; it provides an action plan for when exploitation is discovered, including guidance on protecting victims. It recommends that firms assess the prices paid to suppliers to ensure they enable the provision of decent wages and safe working conditions, and review internal purchasing practices (e.g., recruitment fees, short-term contracts, and sudden workload changes). These provisions are presented as “implementation steps” that adopters “tick if done,” and do not represent any binding agreement to change existing policies.

Although the MSIs are directed toward any property and construction business (regardless of geography or size), almost all adopters are large businesses with international operations and supporters of the management issue frame. Corporate “champions” have been part of several MSIs; these firms’ representatives expressed support for the decent work frame. However, there was no collective engagement from the majority of businesses to integrate the stipulated actions suggested by the decent work frame.

## Discussion

**Fig. 1:**
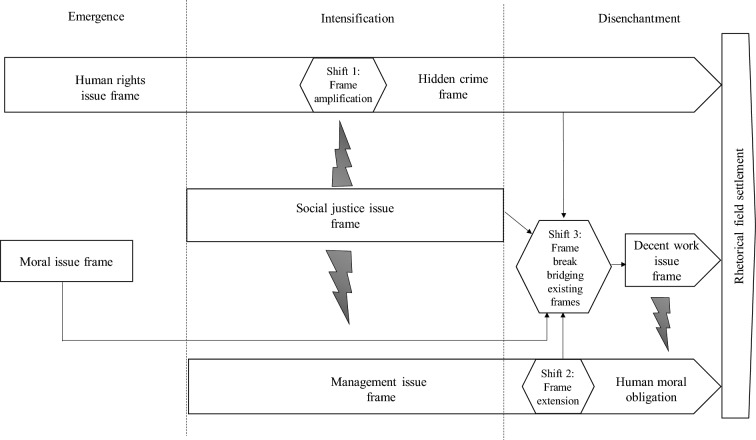
Evolution of the ethical framing of modern slavery and emergence of rhetorical field settlement. Frames appear in rectangular boxes; frame realignments appear in hexagons. Counter-framing activity is depicted with lightning arrows, pointing to the target of the counter-frame. Solid arrows represent the movement of field actors supporting the frame

We now summarize our findings, depicted in Fig. 1, and unpack the significance of the framing dynamics of the ethical issue of modern slavery in the emergence of rhetorical field settlement in the UK construction sector.

Our first observation is that the term “modern slavery” is highly contested in the UK construction sector, mirroring current definitional debates in the academic literature. We empirically identify and track the evolution of five frames—human rights issue (later shifting to hidden crime), moral issue, management issue (later shifting to human moral obligation), social justice issue, and decent work issue—constructed by field actors. Within some of these frames, field actors actively support the labeling of modern slavery, seeking to advance specific interest. The proponents of other frames categorically reject this stance, on the grounds that it distracts from labor exploitation, and shift attention to other issues, such as decent working conditions. The five frames identified differ substantially in both their attribution of responsibility and their recommendations for action. Moral legitimacy was asserted through the invocation of (1) neoliberal arguments to align solutions to the sector’s economic performance and firms’ maximization of profits; (2) normative superiority of workers’ rights; and (3) the historical legacy of British abolitionism and the country’s “goodness”. However, we also found that advocates of the management issue frame frequently adopted a strategy of amoralization (Crane, 2000) in their frames.

Drawing attention to the dynamics of framing, we note that in the early dominant frames, in which modern slavery is seen as human rights and management issues, businesses are absolved of any moral responsibility. By assigning culpability to criminals or to actorless entities and by calling for long-term approaches that downplay urgency, these two frames hinder the development of businesses’ ethical responses. As the framing contests intensified and criticisms of the MSA mounted, these two frames were amplified and extended to legitimize the normativity of the MSA and government’s structures and to emotionally resonate with potential supporters.

The social justice frame was short-lived. Its supporters challenged the use of the term “modern slavery.” It focused on counter-framing (Benford & Snow, 2000) the moral legitimacy of the management issue and the human rights issue frames (see lightning arrows in Fig. 1). Its advocates did not make any reference to ethical solutions nor identify businesses as the responsible actors but problematized the use of the “modern slavery” term and highlighted dissonances and paradoxes in the diagnosis and prognosis of the earlier frames. This, with the purpose to rectify interpretations of modern slavery, that is, to reinstate the agency of those being trafficked and/or exploited, to avoid solutions that might only serve to increase workers’ vulnerability and to treat modern slavery as a risk to people instead of a risk to business. Most business actors adhered to the management issue frame. However, significant disagreements were recorded in their prognosis, leading to the development of two coalitions that clashed on various issues related to compliance with section 54 of the MSA. By focusing on resolving these internal disputes and delaying responses, businesses diverted field actors’ attention from devising ethical solutions to the actual problem and upheld the adoption of private mechanisms and CSR-based anti-slavery approaches. Both coalitions ultimately focused on protecting the status quo. In the eyes of field actors, the management issue frame was fragmented.

Among all the frames, only “moral issue” called for the integration of ethical values into the decision-making of construction professionals. Although it dissipated after period I, some of its advocates steered the emergence of the “decent work” frame that aimed at disrupting dominant accepted arrangements and the moral order in the field (Seo & Creed, 2002). Despite not referring to ethical practices, the “decent work” frame proposed the adoption of various practices “beyond” a set of minimum legal requirements, thus suggesting that playing by the market rules would not solve the problem. Proponents thus established the connection between precarious working conditions and exploitation and entrapment. The decent work frame thus had several merits. First, it rejected the views that modern slavery occurs in a vacuum and that it can be resolved with “business-as-usual” approaches. Second, it shifted the narrative by focusing on the provision of decent working conditions generally, rather than on tackling slavery as an extreme case. Third, it reconnected businesses’ moral agency to the issue, thus empowering the construction industry as a problem-solver.

The new frame catalyzed support and consensus from non-business actors but it lacked a strong motivational framing to galvanize support from businesses, which had not been directly targeted as responsible. There was limited impetus from the largest contractors and construction companies to enact the decommodification of labor and verticalization, as proposed by the decent work frame, as the implementation of these measures would compromise their already thin profit margins. The frame called for collective engagement, but with no clear plan on how to unite an industry long known to be hostile and adversarial, or on how to implement these solutions.

With few exceptions, field actors were “locked” into one modern slavery frame and unable to switch. This “rigid framing” (Palazzo et al., 2012) complicated interactions with other actors adhering to other frames. As depicted in Fig. 1, counter-framing activity was scattered and sustained by only a few frame advocates. Rigid framing thus limited the possibilities for open debate, the moral deliberation of proposed alternatives, and negotiation between actors, and this ultimately inhibited the emergence of common frameworks of action.

Our analysis indicated that the field settlement that was achieved was subtle and could be categorized as rhetorical: businesses changes were mostly rhetorical adjustments, but did not require “departure from business-as-usual” (Feront & Bertels, 2019 p. 16). At the end of 2019, the exogenous shock of Brexit induced a lack of urgency and a sense of uncertainty that pushed the issue of modern slavery to the background, exacerbated proponents’ struggle to make references to the future and rendered the “decent work” frame unactionable (Nyberg et al., 2020).

## Conclusion

### Theoretical Contributions

First, we contribute toward a dynamic understanding of the meaning making of modern slavery which was enabled by our conceptualization of modern slavery as a socially constructed ethical issue (Caruana, 2018; Dahan & Gittens, 2010). This understanding is dynamic because it temporally captures the trajectories of modern slavery frames (tracing realignments, interactions and frame adherence) and situates the framing debate in relation to developments in the UK public and political context. We show that modern slavery frames are not static, and that both advocates and challengers contribute to the realignment of frames, inducing reinterpretations and changes in practitioners’ adherence to specific frames. This understanding also incorporates a wide variety of framing participants. Thereby we address calls to study modern slavery holistically, considering the connectedness of the myriad actors involved in the development of solutions (New, 2015; Van Buren et al., 2021). By documenting the heterogeneity of frames and capturing the often marginalized frames of peripheral actors (unions, labor activists), our study evidences the multivocality of the issue of modern slavery, albeit with power asymmetries (Soundararajan & Brown, 2016), and redirects attention from the extreme view of modern slavery in the literature that recommends a simple understanding of only the most serious offenses (Caruana, 2018) to a plural, fine-grained understanding of the concept as it is constructed and debated by practitioners.

Second, we draw attention to the politics of frames and framing. The findings presented in this study expose the vested interests of actors using particular frames and framing practices. We show that advocates of the human rights and management issue frames purposefully realigned their frames to regain their credibility, to enhance their already dominant position and to push the debate in the direction of their own frames and interests. The majority of business actors deliberately focused on resolving intra-frame disputes. The lack of a unified goal signaled their unreadiness to take action. Inevitably, this stalled the framing position of businesses, which, at the end of the study period in 2019, had deferred any move in the debate.

Our study has also revealed that some critical voices had been left out of the framing debate and thus marginalized from the deliberative process (Hussain & Moriarty, 2018) while other dominant voices had been disproportionately amplified. When analyzing the range of actors participating in framing, the under-representation of workers in supply chains is evident (Reinecke & Donaghey, 2021a). As mentioned in the methodology section above, we were able to capture the framing of some marginalized groups through specific publications and other outlets. However, at most field-configuring events, these actors did not seem to have a strong representation. These observations suggest that the framing dynamics of modern slavery in the UK construction sector add to the reproduction of power relations among actors, by authorizing certain perspectives and restricting others (Meyer & Höllerer, 2010).

Third, our study explicates a path to rhetorical field settlement (Feront & Bertels, 2019) and outlines the conditions that precipitate a frame break. The early dominance and amplification of overly rigid frames that fail to assume or assign moral responsibility to businesses, the limited counter-framing directed at dominant business actors, intra-frame disagreements among businesses, and the absence of businesses’ critical mass of support for the “decent work” frame forestalled the emergence of new patterns of action to tackle modern slavery. Dissatisfaction with the normativity of existing regulation and fear of public moral judgment gave rise to the emergence of the “decent work” frame, which aimed to disrupt dominant accepted norms in the field and the prevailing moral order. This contribution has implications for the literature on the discursive construction of moral legitimacy (Palazzo & Scherer, 2006; Scherer et al., 2013) which identifies strategies and mechanisms through which single organizations justify their conformance to societal moral expectations (Reuber & Morgan-Thomas, 2019; Štumberger & Golob, 2016) and recent studies that have exposed the moral justifications used by specific groups of actors in discourse and narratives of modern slavery (Christ & Burritt, 2018; Islam & Van Staden, 2021; Meehan & Pinnington, 2021; Vestergaard & Uldam, 2021; Wray-Bliss & Michelson, 2021). By focusing on the debate of a controversial issue characterized by moral multiplexity (Reinecke et al., 2017), we shed light on the interplay of frames underpinned by competing moral justifications and its effects on field-level change. Our study not only unravels the argumentative moral moves that each group actors bring into play but also shows that, in the rhetorical field settlement that emerged, there was no compromise of the prevailing moral order.

### Recommendations and Implications for Practice

Our work focused on the evolution of modern slavery frames over time, but we were able to trace in parallel the debate, assent, administration, and initial stage of the review of the MSA, thus revealing its impact in the UK construction sector. The analysis shows that although the MSA has brought the issue into the spotlight and many actors enthusiastically embraced it, it has not induced businesses to assume responsibility for the creation of, contribution to, or links to modern slavery. Advocates of the management issue frame have debated over the purpose, benefits, and costs of compliance and non-compliance with section 54, but these discussions were largely disconnected from the development of solutions to the core problem. The emergence of the “decent work” frame in itself appears to be a consequence of some practitioners’ loss of confidence in the MSA as a catalyst for change. In the absence of effective regulation on the horizon and the emergence of MSIs that do not indicate a departure from business-as-usual (i.e., recommending “best practices” without any agreement on specific businesses’ commitments), the proponents of the “decent work” frame should build up from their “small wins” (Ferraro et al., 2015) highlighted in the current research. One suggestion is to gear up toward models of supply chain governance that enable democratic worker participation (Reinecke & Donaghey, 2021b) which are essential for establishing decent working conditions.

Construction industry knowledge initiatives have been influential in raising awareness and opening spaces (e.g., organizing field-configuring events) for debate, thus contributing to the adaptation of modern slavery views of field actors. However, our findings evidence that “rigid framing” (Palazzo et al., 2012) is prevalent in the field and heightened among some actors and some voices have been marginalized. Government actors pursued a stagnant narrative focused on demonstrating alignment between government’s public opposition to modern slavery and its political stand on immigration. It is thus crucial that governments are open to alternative voices and engage in critical deliberation of their own and others’ frames in the development of solutions.

Our paper also has broader implications, beyond the UK construction sector. Understanding the various frames at play in the anti-slavery movement and the failure of a substantial field settlement can help practitioners in the anti-slavery movement to work toward bridging the divides between frames. Debates and considerations of panel composition need to empower multivocality (Ferraro et al., 2015) to foster interactions whereby actors are exposed to other frames and thus more open to alter their own interpretation and incorporate elements of other frames (Ansari et al., 2013). This will enable practitioners to work toward field settlement and amalgamate currently independent efforts. Our findings will also help policy-making by enabling a structural understanding of the different frames and their underlying assumptions, providing practitioners with the opportunity to compare and triangulate research evidence from different actors, and to assess which frames individual actors are operating in. Similarly, academic researchers need to be reflective about the frame(s) in which they position their work and the positioning of participants in empirical research in relation to those frames.

### Limitations and Future Work

The usual caution in making generalizations is clearly warranted, given the empirical data from a single, revelatory case study. We nevertheless suspect that other fields with centralized structures (Furnari, 2018) (e.g., the tourist industry and project-based industries such as defense) may be susceptible to similar modern slavery framing dynamics as those exposed here. However, this will be also contingent on the political context. As presented in our study, the UK government and the MSA played a significant role in the framing of modern slavery and set the tone. Other countries have opted for “hard” due-diligence law, establishing legally binding obligations for large companies to identify and prevent human rights abuse in their supply chains, which will significantly influence the way businesses and other actors perceive the problem and their responsibilities. More comparative research on modern slavery framing in other sectors with different structures and countries with different legislative approaches is required to further our understanding of the conditions and dynamics of framing.

Although our study covers a six-year period, our interview data were mostly collected at the beginning and at the end of the period, nevertheless, our documentary and participant observation data extensively cover the whole period. Large businesses may be over-represented in our study, partly because the MSA imposes reporting requirements only on large organizations and because of the centralized structure of the field. SME representatives we encountered at field-configuring events were in a minority among business actors. Future studies should pay attention to these and other marginalized actors mentioned in the paper and investigate their own modern slavery framings.

As indicated at the beginning, identifying the origin of actors’ frames is beyond the scope of this paper but our analysis suggests frames’ connections to underlying ideologies^9^ (Creed et al., 2002; Steinberg, 1998). However, for most of the frames this was not clear-cut. Often frames amalgamate elements from more than one ideology and display contradictions. More research could disentangle the discrepancies within frames and the strategic opportunities these paradoxes provide.

Future work adopting a similar longitudinal approach may uncover whether rhetorical field settlement will maintain or disrupt accepted systems of interpretation within the field. Our dataset allows only speculation on whether the “decent work” frame will be amplified in the field and lead to the adoption of new rules and standards. Future studies should examine the process of “solidification” (Nyberg et al., 2020; Reinecke & Ansari, 2016) of modern slavery frames and investigate whether businesses eventually assume responsibility and experiment with any of the proposed solutions.

Our data collection ended in December 2019. On January 31, 2020, the UK left the EU and the COVID-19 pandemic put the world on stand-by in terms of the anti-slavery movement. Future research should investigate the impact of these events on the framing dynamics.

## Supplementary Information

Below is the link to the electronic supplementary material.

Supplementary file1 (DOCX 35 KB)

## Footnotes

1 The terms “contested” and “contentious” are both used in the literature to refer to ethical issues. In this paper we use the latter.

2 Scholars have debated the connection between ideologies and frames. We adhere to Snow & Benford’s (2000) view that ideologies constitute cultural resources that can be tapped and exploited for the purpose of framing. This assumption has methodological implications. Researchers need to assess empirically the nuanced differences in framing rather than simply assuming that frames are coherent packages of ideologies (see Creed et al., 2002; Snow & Benford, 2000).

3 The traditional view of fields considers that fields are constructed around physical proximity and similarity (Wooten & Hoffmann, 2017). Organizational field is commonly defined as a collection of ‘organizations that, in the aggregate, constitute a recognized area of institutional life: key suppliers, resource and product consumers, regulatory agencies, and other organizations that produce similar services or products’ (DiMaggio & Powell, 1983, p.148). An issue-field, in contrast, is more than a collection of a community of organizations, it is the center of common channels of dialog and discussion (Hoffmann, 1999) and thus diverges from the dominant definition in the literature. As a consequence, issue fields typically contain the most diverse set of actors.

4 Ethical approval for the research protocol was granted in that year by the research committee of the lead institution.

5 Some field notes that were not cross-checked were used only to contextualize our analysis.

6 Most of these businesses were subject to section 54 of the MSA.

7 One report was published by the National Audit Office and the other by Her Majesty’s Inspectorate of Constabulary and Fire & Rescue Services.

8 Along with these industry MSIs, the International Standard for Sustainable Procurement BS ISO 20400 provides guidelines for integrating sustainable procurement principles in the organization.

9 The human rights issue frame reflects elements of British political conservatism. The moral issue frame upholds principles of idealism ethical theory (Forsyth, 1992) and evokes elements of neoliberalism. Implicit in the management issue frame is a neoliberal ideology and shareholder value maximization (Djelic & Etchanchu, 2017), while social justice frame bespeaks principles of socialism and communism. The holistic frame in turn evokes premises of the so-called ‘conscious capitalism’ (Mackey, 2011) and principles of decent work (Ghai, 2003).
