# Supplementary material for: Change in Rhetoric but not in Action? Framing of the Ethical Issue of Modern Slavery in a UK Sector at High Risk of Labor Exploitation
Source: J Bus Ethics. 2021 Dec 7;182(1):35–58. doi: 10.1007/s10551-021-05013-w (PMC8649994; doi:10.1007/s10551-021-05013-w)
Supplement: Supplementary file 1 — Supplementary file1 (DOCX 35 KB) [file 10551_2021_5013_MOESM1_ESM.docx]

| **Construction businesses** | Client and construction management companies, general contractors, subcontractors, material suppliers, renovation contractors, equipment lessors, engineering firms, design firms, real estate developers. |
| --- | --- |
| **Industry Skills and standards Body** | Construction Industry Training Board (CITB), The British Standards Institute (BSI) |
| **Certification bodies** | UK Certification Authority for Reinforcing Steels (CARES) |
| **Industry knowledge initiatives** | Supply Chain School, Constructing Excellence, Build UK, Building Research Establishment Group (BRE), APRES, Sustain Worldwide, Construction Sector Transparency Initiative and Engineers Against Poverty |
| **Trade and professional associations** | Chartered Institute of Building (CIOB), Royal Institute of Architects (RIBA), Royal Institution of Chartered Surveyors (RICS), Institute of Environmental Management and Assessment (IEMA), Institution of Civil Engineers (ICE), Chartered Institute of Procurement and Supply (CIPS), Construction Industry Council (CIC), Association of Labour Providers (ALP) |
| **NGOs** | Unseen, Stop the Traffik, Anti-Slavery International, Hope for Justice, Human Rights Watch, The Connection at St Martin in the Fields, The Passage in Westminster. |
| **Campaign groups** | The Blood bricks coalition, Focus on Labour Exploitation (FLEX) |
| **Think tanks** | Business and Human Rights Resource Centre, Institute for Human Rights and Businesses. |
| **Industry Unions** | General, Municipal, Boilermakers and Allied Trade Union (GMB), Unite the Union, UK’s Union of Construction Allied Trades and Technicians (UCATT), International Trade Union Confederation (ITUC), Union Solidarity International (USI). |
| **Law enforcement and government agencies** | The Home Office, Modern Slavery and Kidnap Unit, the Metropolitan Police, the Gangmasters and Labour Abuse Authority (GLAA), The National Crime Agency. |
| **Watchdogs** | The Anti-slavery commissioner, Joint Committee on Human Rights, Labour Exploitation Advisory Group, Corporate Responsibility Coalition (CORE) |
| **Government figures** | Members of Parliament, Minister with responsibility for Building Regulations, Minister for Crime, Safeguarding and Vulnerability, Minister for Modern Slavery and Organised Crime, Minister for Trade and Investment. Business Minister for Employment Relations, Baroness Young of Hornsey, UK Home Office Minister, Minister for safeguarding, vulnerability and countering extremism, the Independent Anti-slavery Commissioner, the Home Affairs Select Committee |
| **Consultancy firms** | Action Sustainability, Resilient World, BSI supply chain, PwC, Ergon Associates, Mazars LL, EY, Thompson solicitors, Responsible Trade Word Wide, Upstream Sustainability Services at Jones Lang LaSalle, Gowling |

**Table S1. Full list of field actors**

**Table S2. Timeline of Selected Events**

| **Date** | **Actor** | **Event description** |
| --- | --- | --- |
| 2014  2015  2016  2017  2018  2019 | UNION  UNION  MEDIA  NGO  CAMPAIGN GROUP  FIRM  POLICE  UKGOV  POLICE  UKGOV  NGO  VICTIMS  FIRMS  GOV AGENCY  PROF ASSOC  PROF ASSOC  NGO  FIRMS  MEDIA  INDUSTRY INITIATIVE  WATCHDOG  CAMPAIGNERS  FIRMS  UKGOV  POLICE WATCHDOG  VARIOUS  VARIOUS  NATIONAL AUDIT OFFICE  GOV AGENCY  FIRM  VARIOUS  INDUSTRY INITIATIVE  UN  POLICE  CONSULTANCY  GOV AGENCY  POLICE  GOVUK  GOVUK  GOVUK  GOVUK  COURT | ITUC report: ‘*The case against’ Qatar*  UCATT unveils exploitation in Qatar.  BBC’s Newsnight investigation of immigrant exploitation in Qatar.  Amnesty International’s report ‘*Promising little, delivering less’* on Qatar’s proposed reforms to tackle modern slavery.  The Blood Bricks coalition exposes cases of bonded labor in India involving Larsen and Toubro and the Howden Group.  West Midlands Police finds Romanians subjected to forced labor in construction in Dudley.  MSA section 54 comes into force.  West Midlands Police finds modern slavery cases of construction workers.  Government outlines proposals to reform the powers and remit of the Gangmasters Licensing Authority (GLA).  Amnesty International’s report: ‘*The ugly side of the beautiful game: Exploitation on a Qatar 2022 World Cup site’.*  Allegations of exploitation of workers on Doha construction sites involving Balfour Beatty and Interserve.  GLAA obtains new powers to investigate labor abuse.  CIOB report: ‘*Building a fairer system: tackling modern slavery in construction supply chain.’*  CIOB and Stronger Together launch a best practice toolkit to tackle modern slavery.  First wave of modern slavery statements published.  Lexis Nexis BIS’ report: ‘*Modern slavery in the Construction Industry.’*  First Modern Slavery and Ethical Labour in Construction Leadership Symposium.  CORE report highlights deficiencies in compliance with MSA section 54.  Demonstrations following accusation of exploitation of non-UK workers by CNIM and Wheelabrator.  First reading of the Transparency in Supply Chains Bill in the House of Lords, seeking to amend existing requirements.  Publication of report ‘*Stolen Freedom: The policing Response to Modern Slavery and Human Trafficking.’*  London Mayor endorses London ‘*Evening Standard* campaign on modern slavery.’  Businesses, law firms, the Met, the City of London Corporation, and the Confederation of British Industry sign up to the Evening Standard’s “*Stop Slavery Pledge*.”  Publication of report ‘*Reducing Modern Slavery.’*  Photographic exhibition ‘*Invisible People’* presented as part of the National Crime Agency’s Modern Slavery and Human Trafficking campaign.  Kwik Structures accused of exploiting workers.  10 UK businesses sign up to Bright Future, a scheme devised by the Co-op and City Hearts charity.  BRE launches the 2018 Annual UK Top100 Corporate Modern Slavery Influencers’ Index.  UN Human Rights Council publishes the ‘Zero Draft’ Treaty.  Reports show continuous rise of police prosecutions of trafficking crimes.  Ergon Associate’s report: Modern slavery reporting: Is there evidence of progress?  GLAA launches the Construction Protocol.  Bradford men convicted on modern slavery charges after 2013 West Yorkshire Police raid.  Appointment of new IASC  Home Office publishes refreshed guidance on the reporting requirements of MSA section 54.  Independent review of the MSA is published.  Government responds to recommendations of the independent review of MSA.  Romanian organized crime group sentenced for trafficking victims to exploit them within the construction industry. |

**Table S3. Illustrative quotes of the three framing functions**

| **Punctuation**  **function** | | “This is the great human right’s issue of our time… I am determined that we will make it a national and international mission to rid our world of this barbaric evil” (Prime Minister, The Telegraph, 30 July 2016).  “We took evidence from police officers, prosecutors and regulators, and from non-government organizations who support rescued slaves. They all made the same point - slavery is often hidden in plain view” (MP, Building Magazine, 12 December 2014).  “It is accepted by most civilized societies that utilizing forced, bonded or trafficked labour is unethical, inhumane and evidence of the worst aspects of human nature” (Sustainable Procurement and Modern Slavery Senior Consultant, 2016).  “Those in construction are especially vulnerable to this crime; with high demand for low wage labour, we must therefore strive to see a thriving construction industry that values ethical recruitment and fair employment if we ever hope to end this evil trade in human beings” (Former Independent Anti-Slavery Commissioner, Lexis Nexis report, 5 September 2016).  “Low-level abuses were frequently a precursor to more offences, and more serious offences” (British trade union, Labour Research, 25 February 2016.  “Relaxing the licensing regime would be as useful as a chocolate fireguard in preventing exploitation in industries like construction” (Acting secretary of a construction workers’ union, Labour Research, 25 February 2016).  “We must think way beyond slavery and not accept exploiting people full stop, because slavery is at the extreme end of a spectrum of exploitation” (CEO, International NGO against slavery, The Independent, 17 April 2019).  "Human rights are absolutely universal and therefore, whether somebody is in squalid conditions here or in other parts of the world and having to work a ridiculous number of hours, that is something which we should be concerned about and acting to change in which responsible UK companies will act to change'' (MP, The Press Association, December 16, 2014). |
| --- | --- | --- |
| **Elaboration function** | **Diagnostic**  **framing** | “The construction industry has become an easy target for unscrupulous slave masters seeking to place their products” (Representative of the Department for Business, Innovation and Skills, Building Magazine 12 December 2014).  “While this data gives a good snapshot of what kinds of slavery are most prevalent and who is falling victim to exploiters, it doesn't paint the whole picture. For every victim identified by the police, there will be many others who are not found and remain under the control of traffickers, pimps and Gangmasters” (Human Rights Journalist, The Guardian, 18 October 2018).  “The issue with the construction sector seems to be that it operates reactively when it comes to huge ethical issues such as crime, corruption and modern slavery” (Public Affairs Manager at a Construction professional body, Building Magazine 15 May 2019).  “The system is fueled by factors such as debilitating poverty and discrimination, and reinforced by a system that ignores, and blames victims. It's a potent formula” (CEO, International NGO against slavery, The Independent,17 April 2019).  “The industry is desperate for labour, so you have contractors either not checking or failing to check properly. This could mean allowing staff without sufficient training to conduct checks or outsourcing the checking to the organization supplying the labour which could be a criminal gang” (CEO at a Construction professional body, Building Magazine 28 August 2015).  “The widespread and everyday experience of exploitation is not due to organised criminal gangs, but due to unscrupulous employers who do not observe minimum standards and who are not committed to providing decent pay and decent working conditions for their employees" (Representative of a trade union, Labour Research, 25 February 2016).  “Until the unnecessarily long labour supply chains are tackled the potential of modern-day slavery will exist in every area of our industry” (Officer of a British trade union, 21 December 2019). |
|  | **Prognostic**  **framing** | “Companies should have to report on their working conditions and that of their suppliers to ensure that we have the transparency in our supply chains and can help reduce the risk of modern slavery” (MP, The Press Association, 16 December 2014).  “One of the things that we place at the centre of our modern slavery response is the victim. A victim centered response, that may be seen as an odd thing for a corporate to say. I’m sure there are other NGOs in the room who say that’s perhaps their remit but we put the victim firmly at the centre of our response” (Business and Human Rights Manager at a large materials company, field configuring event, 18 October, 2017).  “Firstly, get to grips with the supply chain and understand where the labour is coming from. Mapping a company's operations will help identify where the material risks are and who to collaborate with to prevent forced labour. Develop human rights and supply chain policies, to communicate the company's commitments to ensure a slavery free supply chain and embed these into procurement processes. A policy should be supported by due diligence to monitor compliance, and set out what to do in the event that instances of slavery are uncovered” (Sustainability Consultant, Building Magazine, 22 December 2015).  “What we encourage businesses to do is have a robust recruitment process ... and encourage an open dialogue with the labour workforce so you can build that trust and get them to open up [if they are victims of modern slavery]” (Representative of the Gangmasters Licensing Authority, Building Magazine, 16 November, 2016).  “Slavery, anywhere, must not be tolerated in the 21st century, and our work to stamp out this practice abroad will support our effort to end slavery in the UK. This is a long-term challenge and others must follow our lead” (Member of the British Cabinet, Financial Wire, 01 December 2017).  “We are committed to ensuring there is no modern slavery in any part of our business or supply chain, and we are implementing and enforcing effective systems and controls to enforce our approach, extending to our own employees and the thousands more employed in our supply chains, including sub-contractors, suppliers and labour agencies” (Acting CEO, of Family owned construction company, The Evening Standard, 24 November 2017).  “Organizations even those not required by statute to publish a slavery and human trafficking statement - should be encouraged to continue to consider and address the issue of modern slavery within their supply chains if they wish to win new work and avoid potentially severe brand damage” (Senior Solicitor, International Law firm, Mondaq Business Briefing, 23 February 2017). |
| **Motivation function** | | “The UK has published our action plan and there are now a handful of other countries in the world that have done so, but we need to make sure that we are using that leadership as the UK to do the things we need to do in our own country and to encourage other countries to do this as well” (MP, The Press Association, 16 December 2014).  “Following Carillion, Grenfell and everything else, the industry is really under the microscope. The number one priority of BEIS is to change the construction industry's business model. For construction, the prospect of further CMA action - with the latest probe launched just in March - will not be welcome. But cleaning up its act seems likely to be just one of a number of necessary measures to reform the industry as a whole” (Public Affairs Manager, CIOB, Building Magazine, 15 May 2017).  “Marking our 180th anniversary puts ending slavery in a wider perspective and acts as a reminder that ending slavery will take time - any attempts to cut corners will end in failure. But it also reminds us that whatever the task, we can solve this problem with every person freed, every law and policy changed, bringing us a step closer to a world without slavery even if that aim looks distant now” (Representative international NGO against slavery, The Independent,17 April 2019). |
